# Modifying upper-limb inter-joint coordination in healthy subjects by training with a robotic exoskeleton

**DOI:** 10.1186/s12984-017-0254-x

**Published:** 2017-06-12

**Authors:** Tommaso Proietti, Emmanuel Guigon, Agnès Roby-Brami, Nathanaël Jarrassé

**Affiliations:** Sorbonne Universités, UPMC Univ. Paris 06, CNRS, UMR 7222, INSERM, the Institute of Intelligent Systems and Robotics (ISIR), 4 place Jussieu, Paris, 75005 France

**Keywords:** Upper-limb robotic exoskeletons, Rehabilitation robotics, Motor coordination learning, Force fields adaptation, Motor redundancy

## Abstract

**Background:**

The possibility to modify the usually pathological patterns of coordination of the upper-limb in stroke survivors remains a central issue and an open question for neurorehabilitation. Despite robot-led physical training could potentially improve the motor recovery of hemiparetic patients, most of the state-of-the-art studies addressing motor control learning, with artificial virtual force fields, only focused on the end-effector kinematic adaptation, by using planar devices. Clearly, an interesting aspect of studying 3D movements with a robotic exoskeleton, is the possibility to investigate the way the human central nervous system deals with the natural upper-limb redundancy for common activities like pointing or tracking tasks.

**Methods:**

We asked twenty healthy participants to perform 3D pointing or tracking tasks under the effect of inter-joint velocity dependant perturbing force fields, applied directly at the joint level by a 4-DOF robotic arm exoskeleton. These fields perturbed the human natural inter-joint coordination but did not constrain directly the end-effector movements and thus subjects capability to perform the tasks. As a consequence, while the participants focused on the achievement of the task, we unexplicitly modified their natural upper-limb coordination strategy. We studied the force fields direct effect on pointing movements towards 8 targets placed in the 3D peripersonal space, and we also considered potential generalizations on 4 distinct other targets. Post-effects were studied after the removal of the force fields (wash-out and follow up). These effects were quantified by a kinematic analysis of the pointing movements at both end-point and joint levels, and by a measure of the final postures. At the same time, we analysed the natural inter-joint coordination through PCA.

**Results:**

During the exposition to the perturbative fields, we observed modifications of the subjects movement kinematics at every level (joints, end-effector, and inter-joint coordination). Adaptation was evidenced by a partial decrease of the movement deviations due to the fields, during the repetitions, but it occurred only on 21% of the motions. Nonetheless post-effects were observed in 86% of cases during the wash-out and follow up periods (right after the removal of the perturbation by the fields and after 30 minutes of being detached from the exoskeleton). Important inter-individual differences were observed but with small variability within subjects. In particular, a group of subjects showed an over-shoot with respect to the original unexposed trajectories (in 30% of cases), but the most frequent consequence (in 55% of cases) was the partial persistence of the modified upper-limb coordination, adopted at the time of the perturbation. Temporal and spatial generalizations were also evidenced by the deviation of the movement trajectories, both at the end-effector and at the intermediate joints and the modification of the final pointing postures towards targets which were never exposed to any field.

**Conclusions:**

Such results are the first quantified characterization of the effects of modification of the upper-limb coordination in healthy subjects, by imposing modification through viscous force fields distributed at the joint level, and could pave the way towards opportunities to rehabilitate pathological arm synergies with robots.

**Electronic supplementary material:**

The online version of this article (doi:10.1186/s12984-017-0254-x) contains supplementary material, which is available to authorized users.

## Background

Post-stroke hemiparetic patients generally exhibit pathological synergies in their upper-limb movements, resulting in global and stereotyped unnatural patterns of coordination of their arm joints. These impaired synergies can induce in the stroke survivors harmful compensations at the shoulder and trunk level, having a negative impact on the quality of movement performance and potentially limiting the long term prognosis [1, 2]. Therefore, a central question in neurorehabilitation is whether or not it is possible to modify the patients impaired upper-limb synergy in order to regain a more natural and healthier control of the arm. The mechanisms of recovery after stroke are multifactorial and the quantification of the effect of rehabilitation programs is complex [3], but it is known that physical training can lead to permanent improvements in motor function on patients with motor deficits [4].

Since the mid 90s rehabilitation robotics has arisen as one possible solution to provide intensive goal-directed assisted training, with potentiality to improve human-led therapy [5]. For this reason many robotic devices for rehabilitation have been recently developed [6]; however, despite the large number of robotic exoskeletons, the studies addressing motor control learning, exploiting artificial force fields, were mostly performed using planar robot or manipulanda [7], i.e. robots with maximum two Degrees Of Freedom (DOF), thus addressing end-effector 2D movements with no control on and interaction with the other upper-limb joints.

Among these, a landmark result on motor learning was given by Shadmehr and Mussa-Ivaldi in 1994 [8]. Several healthy subjects were asked to perform straight-line point-to-point motions, under the effect of velocity-dependent deviating force fields, produced by a planar robot. After an initial failure phase, due to the deviating fields, the users were able to learn how to complete the required task by adapting to these disturbances. The term *adaptation* describes the progressive lessening of the effect of the perturbation performed on the human upper-limb movements by the presence of the force fields. Once the force fields were removed, participants temporary showed an over-shoot on the opposite direction of the fields, as an *after-effects* or *post-effects*. Authors explained this phenomenon as the generation, by the human Central Nervous System (CNS), of an *internal model* of the disturbances produced by the fields, which was utilized to compensate the deviating forces in a feedforward manner.

*Learning* is quite a complex process, concerning the presence of several phenomena such as motor adaptation, post-effects of this adaptation, spatial generalization, and temporal retaining of these effects. In particular, what is crucial for modern neurorehabilitation is the aptitude of the impaired patients to transfer the post-effects to different activities of daily living (*generalization*), and to maintain these effects during the weeks after the training (*retaining*).

Based on the internal model hypothesis and by adopting similar experimental setup, others successive studies were carried out, providing evidence that motor adaptation is influenced simultaneously by dynamic and kinematic factors [9], and by the presence of visual feedback on the error [10]. Nonetheless, participants were even able to transfer post-effects in regions of the workspace where no exposure to the force fields took place [8, 10].

These studies were all limited to movements in a plane by the architectural properties of the utilized devices. Moreover, due to the limitations of the planar devices, they rarely directly addressed intermediate joint movements and inter-joint coordination analysis. Dipietro et al. [11], for example, showed how the shoulder-elbow impaired independence was improved in a circle drawing task for 117 chronic stroke survivors, after training with a planar robot on a therapy of assisted point-to-point movements. However, the shoulder and elbow joint angles were only deducted from the measured hand path, through a simplified non-redundant two-link model of the human arm.

Modern robotic exoskeletons, on the contrary, thanks to their 3D structures and a larger number of DOF with respect to planar robots, are able to impose forces at each different joint of the upper-limb separately and to collect reliable measures of the intermediate joints movements, providing a promising framework to test adaptation and learning on 3D activities. Clearly, an interesting aspect of working with exoskeletons, is the possibility to investigate the way the human CNS deals with the natural upper-limb redundancy for common activities like pointing or tracking tasks. Indeed the CNS is known to exploit synergies, i.e. fundamental building blocks of the motor control [12], to couple joints or muscles movements together, in order to decrease and face the redundancy of the system [13]. Despite the large redundancy of the upper-limbs, the final posture, reaching a given position in the space, is quite reproducible for each individual subject [14]. However the factors that determine these final postures remain disputed, in particular the relative importance of static versus dynamic constraints ([15–17], see [18] for a review).

We are interested in better understanding the possibility of former results on manipulanda to be generalized to inter-joint coordination in such a redundant system, i.e. the 7-DOF of the human arm. In fact, beyond performing therapy for upper-limb movements and functional recovery, there is a growing need for rehabilitating synergies of patients towards more normal patterns of motion, but yet fundamental studies and experimental results are lacking on how humans, even healthy, adapt to constraints imposed on the inter-joint coordination.

To the authors’ knowledge, only Mistry et al. [19] preliminary investigated human force field adaptation using an exoskeleton with multiple coupled parts and joint level interactions (a 7-DOF *Sarcos Master Arm* robotic exoskeleton). However, the authors of this experiment limited the application of the force fields to a single joint; in particular, they used the exoskeleton to perturb the elbow flexion/extension, during point-to-point reaching tasks, by driving a disturbing force based on the shoulder velocity (respectively the sum of the shoulder flexion/extension and the shoulder abduction/adduction). The authors found that the human nervous system exploited the redundancy of the upper-limb to minimize the effects of the force field on the realization of the endpoint trajectory. In fact, and after a period of adaptation to the force field (about 100 movements) the hand trajectories returned to baseline but the trajectories at the joint level remained changed. After-effects were present after the removal of the perturbation; however this result was very preliminary since it consisted of only 3 catch-trial movements, with no analysis of the wash-out.

The goal of this research is therefore to try to answer the following questions by utilizing a robotic exoskeleton. Can we modify the upper-limb synergy and teach a specific inter-joint coordination? If we apply some viscous distributed inter-joint constraints through an exoskeleton, without constraining the end-effector movements, how would subjects react? Would participants, exploiting the upper-limb redundancy in the perturbing environment, adapt their motor coordination and show similar after-effects as the ones appearing on the end-effector in state-of-the-art studies, in which, however, the effects on the inter-joint coordination were not taken into consideration?

Such knowledge could be fundamental to exploit the main characteristics of modern robotic exoskeletons – the possibility to control multiple joints separately, by imposing distributed coordination control – for neurorehabilitation and could lead to the opportunity, in a near future, to rehabilitate pathological synergies with robots.

## Methods

### Participants

Twenty healthy individuals participated in this study (12 male and 8 female, aged 24.5±4.8). They were members of the laboratory or relatives, naive to the experiment. The experimental protocol has been validated by the ethics committee of the Paris Decartes University and the participants gave informed consent before participation. We measured subjects size and their maximum grip force with a dynamometer.

### Instrumentation : ABLE exoskeleton

**Fig. 1:**
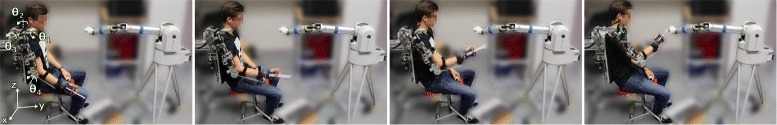
Example of goal-directed pointing task (GDM). The four pictures show the motion from the starting position to the WAM button, while performing GDM task. In this case the subjects were not asked to follow any specific endpoint trajectory

ABLE is a right-arm robotic exoskeleton designed by the CEA-LIST [20], a four active DOF robot, with 3-DOF at the shoulder (for abduction/adduction *θ*_1_, internal/external rotation *θ*_2_, and flexion/extension *θ*_3_) and one at the elbow (for flexion/extension *θ*_4_), see Fig. 1.

ABLE has interesting features for rehabilitation robotics, that are large workspace (it allows about 110° of rotation at the first three shoulder axes, and 130° at the elbow), force/torque ranges compatible with human ones (18Nm available on the first two joints, 13Nm on the last two, producing an equivalent maximum force at the hand of 50N), and above all high backdriveability, thanks to a patented screw-cable mechanical transmission [21].

While the human joint kinematics are very complex and cannot be perfectly imitated by conventional robotic joints (principally because simple robotic pivot joints with fixed rotational axes can not reproduce the complex geometry of interacting bone surfaces which modify rotation axes during the joint displacements; but also because there is yet no consensual model of human kinematics for certain joint group, like the shoulder scapula-clavicle group) we consider in this paper that the similarity between the human and robot kinematic chains is high enough for this kinematic discrepancies not to perturb the subject behaviour. This is also made possible by the compliance of the fixations used and of the human tissues which limit the overall hyperstaticity consequence (i.e. the appearance of uncontrolled forces). Therefore in this study we consider a direct equivalence between the robot and the human joints, and used the exoskeleton encoders as the measurement source of the human kinematics.

### ABLE controllers

The control algorithms were coded on a real time controller (RTLinux running control loop at 1kHz). We utilized two different control modes on the ABLE exoskeleton: the *Gravity Compensation* mode, to allow unconstrained upper-limb motion, and the *Kinematic Synergy Control* (KSC), to expose subjects to the corrective force field.

#### Gravity Compensation

This control mode consisted in a feedforward full gravity compensation of the exoskeleton (weight about 13Kg). With this mode, the robot produces minimal resistance to the human motion, giving freedom of movement to the user [22]. Actually, the gravity compensation was always active as a feedforward compensation, even simultaneously to the KSC, thus this control mode can be seen as the situation in which the perturbing force fields were inactive.

#### Kinematic Synergy Control

The Kinematic Synergy Control (KSC) is a controller, developed by Crocher et al. [23], which generates reactive viscous joint torques to impose specific patterns of inter-joint coordination without constraining the hand motion in space. By exploiting the human natural upper-limb redundancy when performing reaching tasks, this controller corrects the free arm movement if the operator is not respecting a desired synergy – a desired ratio among the upper-limb joint velocities – thus encouraging the user to change his inter-joint coordination. In this case, the KSC generates dissipative velocity dependant forces to constrain the undesired upper-limb coordination directly at the joint level, leaving freedom to the end-point motion. Otherwise, if the given pattern of joint coordination is followed, the controller produces a null torque.

For our experiment we used the following version of the KSC:

1 $$ \tau = -k\mathbf{C}^{T}\mathbf{C}\dot{\mathbf{q}}   $$

where *τ* is the vector of the output torque to the exoskeleton joints *τ*=[*τ*_1_ *τ*_2_ *τ*_3_ *τ*_4_]^*T*^, $k \in \mathbb {R}^{4} $ is a vector of viscous gains, $\dot {\mathbf{q}} \in \mathbb {R}^{4} $ is the vector of joints velocity, and $\mathbf{C} \in \mathbb {R}^{4} $ is an arbitrary imposed vector of constraints, which we set for the experiment as

2 $$ \mathbf{C} = \left[ 0.667 \quad 0 \quad -0.667 \quad 0.333 \right].   $$

The chosen values of **C** provided a generic perturbing behaviour pushing towards unnatural inter-joint coordination, as, for example in our experiment over abduction while flexing the shoulder during forward hand movement, instead of the natural synergy (shoulder flexion and elbow extension). Furthermore, the resulting constraints, imposing a ratio among the different joint velocities, were complex for the participants to be understood, providing a not easily predictable exoskeleton behaviour for the users.

### Experimental setup

#### Tasks

The participants were asked to perform several pointing tasks within the ABLE exoskeleton, while sitting comfortably on a stool. The exoskeleton was connected to the right arm of the operator through three velcro cuffs, one on the upper-arm and two on the forearm. Besides, the subjects wore a commercial wrist splint to limit wrist motion and prono-supination (not controlled nor measured by the robot).

A 7-DOF WAM manipulator (Ⓒ *Barrett Technology*), with a press button at its extremity, was placed in front of the participants for presenting the pointing targets. Starting from a resting position (the upper-arm along the body, with the elbow bent about 90 degrees and the forearm along the leg), the participants were asked to push the button on the WAM through a plastic rod, screwed on the splint. Once the target button was pressed, the ABLE exoskeleton was actively bringing back the upper-limb to the starting position, through a position control mode, while the subjects were remaining passive. For each pointing trial, participants manually triggered the record of the motion by pushing on a secondary button with the free left hand and they had 4s to complete the movement.

**Fig. 2:**
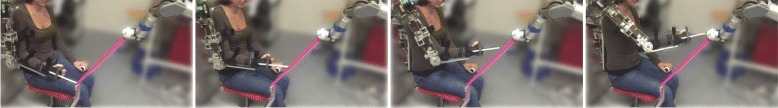
Example of path-constrained tracking task (PCT). The four pictures show the motion from the starting position to the WAM button, while performing PCT exercise. The participants were asked to follow the specific endpoint path shown by the rubber band going from the starting position of the ABLE exoskeleton to the WAM end-effector

We chose two different testing tasks, after a preliminary experiment reported in Additional file 1:

- *Goal-Directed Mode* (GDM), a simple pointing task, the participants were free to move from the starting position to the button, i.e. they were not asked to follow any specific trajectory or arm coordination, see Fig. 1.
- *Path-Constrained Tracking mode* (PCT), a path following task, the subjects were asked to follow a specific straight end-point path from the initial position to the WAM push-button, see Fig. 2. The endpoint path was shown by an elastic rubber band connecting the starting position to the WAM button.

The average total times for each reaching task were about 1,5 hours for GDM and 15 minutes longer for PCT. A video of the experiment is presented as Additional file 2.


Additional file 2: Video of the experiment. (MP4 4470 kb)


#### Reaching targets

**Fig. 3:**
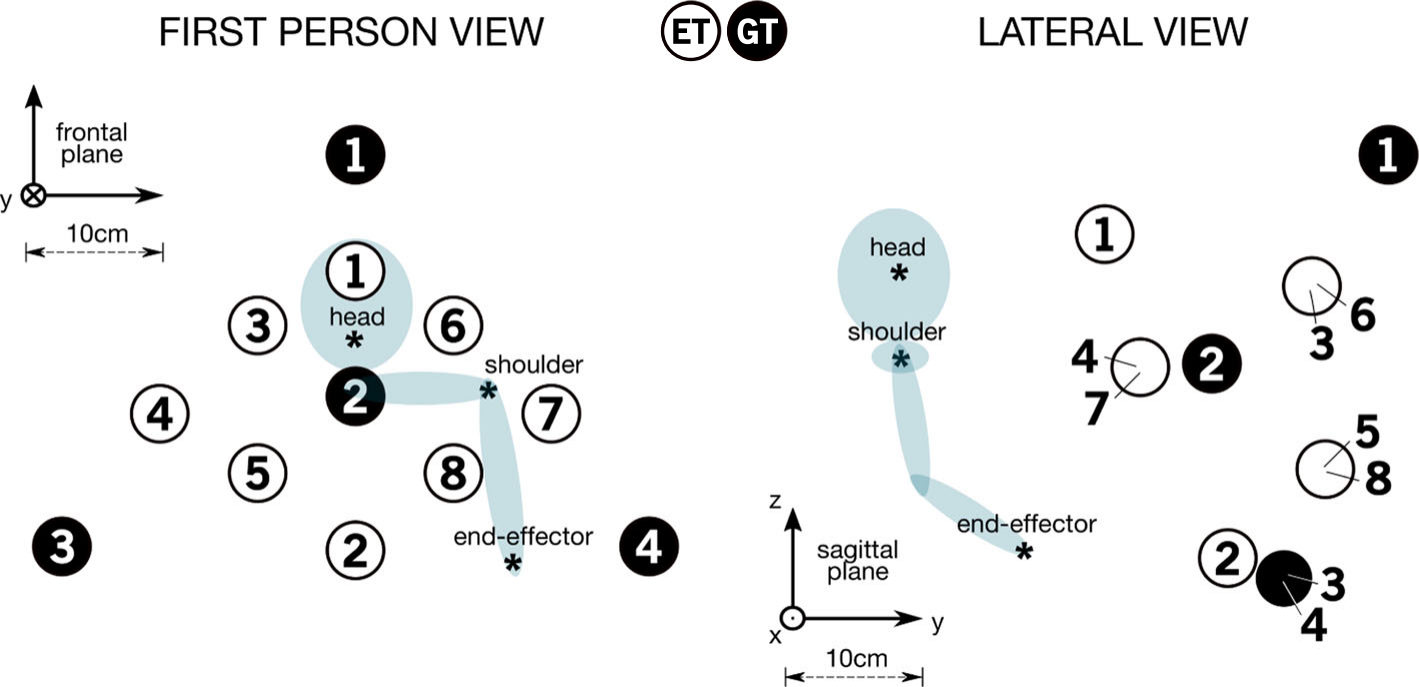
WAM positions. The eight Experimental Target positions (ET) and the four Generalization Target positions (GT). The asterisks * show the mean position of head and shoulder, and the projection of the starting position of the elbow/end-effector. On the *left*, *x-z* frontal plane, on the *right*, *y-z* sagittal plane (some targets are coincident on this plane). The frames are consistent with the reference frame of Fig. 1

The protocol consisted in 12 different final positions presented by the WAM robot, grouped in *Experimental Targets* (ET, 8 positions) and in *Generalization Targets* (GT, 4 positions), shown in Fig. 3. While the ET were periodically exposed to the perturbation of the force fields, the GT positions were never exposed to any field. These positions were fixed for all the subjects. The targets ET 1 and GT 2 were placed in the sagittal plane of the participant, the ET targets 6–8 and ET 7 were placed in a para-sagittal plane respectively more internal and external to the plane of the participant’s upper-arm. The targets were placed on different depth (about 15*c* *m* of range between the closest position to the subject and the furthest one, Fig. 3). The distance WAM-ABLE was fixed for all the participants, but selected such as every participant could reach any target position within the exoskeleton.

#### Procedure

We grouped the participants by ten for attempting one of the two exercises, respectively GDM or PCT. Initially, every subject was given the possibility to practice free movements in gravity compensation mode inside the exoskeleton for few minutes. After this initial training, the subject was asked to point the different positions within the robotic exoskeleton. All the sequences of pointing tasks were performed by blocks of 8 ET or 4 GT trials presented in the same randomized order for each subject. The experiment consisted of 4 phases: *preliminary* (PRE), *experiment* (EXP), *wash-out* (WAS), and *follow up* (FOL). In particular we had:

- **PRE** (24 total pointing task) was 2x8 ET followed by 2x4 GT;
- **EXP** (300 total pointing task) was 15 repetitions of 2x8 ET plus 4 GT, that is every repetition consisted of 20 pointing tasks;
- **WAS** (40 total pointing task) was two repetitions of 2x8 ET plus 4 GT;
- **FOL** (40 total pointing task) was two repetitions of 2x8 ET plus 4 GT.

**Fig. 4:**
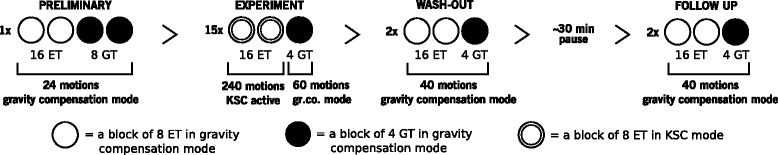
Phases of the experiment. Experimental protocol, showing the four consecutive phases, respectively *preliminary*, *experiment*, *wash-out* and *follow up*. Before the follow up, the subject was resting, detached from the exoskeleton, for about 30 minutes. The number in front of each phase stands for the number of repetition of each pattern (1 repetition for PRE, 15 for EXP, and 2 for WAS and FOL)

Figure 4 shows a scheme of the patterns and the phases.

PRE, EXP, and WAS were performed in sequence, while before FOL there was a pause of about 30 minutes, during which the participants rested, detached from the exoskeleton. It is important to underline that the KSC was active only during EXP and only during the pointing tasks towards ET. Otherwise, the robot was controlled in gravity compensation mode. Therefore, as shown in Fig. 4, the KSC was perturbing and correcting the subject free motion only on 240 motions over the 404 total motion of each experiment. GT movements were always unconstrained motions.

#### Quantification of the human spontaneous variability within the exoskeleton

A specific experiment was performed in order to quantify the spontaneous variability of human subjects performing reaching tasks, within the exoskeleton, in gravity compensation mode. We performed this experiment with 10 healthy subjects – participants of the KSC experiment but naive at the moment of the variability test – performing pointing task to GT targets. Five subjects practised with each exercise (GDM and PCT). They performed four sequences of five repetitions of pointing tasks, towards the 4 GT positions (thus a total of 20 motions per sequence) for a total of 80 reaching tasks for each subject. The sequences were separated each other by one hour of resting time, during which the participants were detached from the exoskeleton.

## Data processing

### Data gathering

Data were directly and only collected through the sensors of the ABLE exoskeleton, without the use of any external motion capture system. The ABLE joint kinematic data were measured through differential encoders placed at the joints level. For the end-point kinematics (the tip of the rod) a direct kinematic model of the robotic upper-limb was used together with a measure of the subject forearm length. Post-processing was done using Matlab environment (Ⓒ *The MathWorks, Inc.*). All the recorded data were passband filtered through a 4th order Butterworth filter (cutoff = 5Hz).

### Joint kinematics

We analysed the final arm-robot configuration at the time when the subjects were pushing the WAM button. The final posture joint angles *q*_*i*_, with *i*=1,2,3,4, were expressed relatively to their values at the end of the first movement in the preliminary phase, thus before any force fields exposition.

### End-effector kinematics

We computed the speed profile (norm of velocity) of the endpoint motion and considered the following temporal parameters

- motion duration *T*, for which we defined the beginning and end of each movement as 10% of its peak velocity;
- end-point motion smoothness *η*, as the spectral arc-length metric defined by [24], for which large negative values mean reduced smoothness;
- maximum end-point velocity *v*_*max*_.

The spatial charateristics of the trajectory were studied thanks to the *curvature* parameter *Φ*, defined by [22] as the maximum deflection of the hand path from a straight line joining the initial and final positions, showing if the hand is deviated from its natural path:

3 $$ \Phi = \frac{1}{l}\max(d_{p}(t))   $$

where $l = \left \| \overrightarrow {P(t_{end})} - \overrightarrow {P(t_{in})} \right \|$ is the value of the straight line from starting to final position, and

4 $$ d_{p}(t)=\frac{1}{l} \left\| \left(\overrightarrow{P(t)} - \overrightarrow{P(t_{in})} \right) \times \left(\overrightarrow{P(t_{end})} - \overrightarrow{P(t_{in})} \right)\right\|  $$

is the instantaneous distance of the vector position of the pointer, $\overrightarrow {P(t)}$, from the straight line joining $\overrightarrow {P(t_{in})}$ and $\overrightarrow {P(t_{end})}$.

### Principal Component Analysis

A Principal Component Analysis (PCA) was performed on the simultaneous coordination of the four joints, *i.e.* a test to spot potential resulting *joint synergy*. Each PCA was performed on the joint velocities of a single repetition of reaching tasks, therefore on four reaching movements for the GT (one PCA for each black circle in Fig. 4) and on sixteen reaching movements for the ET (one PCA for each couple of white circles in Fig. 4).

In order to determine if two motions were similarly coordinated, we applied the metrics previously developed in [25] that is a distance metrics between subspaces defined by the three Principal Components (PCs):

5 $$ \psi\left(\mathbf{U},\mathbf{V}\right) = \sqrt{1-S_{min}^{2}\left(\mathbf{U}^{T}\mathbf{V}\right)}  $$

where *S*_*min*_ is the minimal singular value decomposition of the matrix **U** ^*T*^ **V**, with **U** and **V** the two subspaces. This function represents the sine of the minimal angle of rotation between the two subspaces, where *ψ*=0 stands for no rotation and *ψ*=1 for orthogonality. Thus, a small distance between subspaces, representing different motions, would mean that these motions were performed by using similar joint synergies, while a large distance would indicate that the subject has changed his coordination while performing the same task.

### Statistics

For the statistical analysis on the whole group of participants, the analyses were performed separately for the ET and GT trials. The values of the end-point and the joint variables were averaged during five phases of the procedure, for each target. In particular we considered 2 blocks of PRE trials, the two first and two last blocks of the EXP trials, respectively Early and Late exposition to KSC (E-EXP and L-EXP), the first two blocks of wash-out WAS, and the first two of follow up FOL.

Non-parametric statistics were performed on these five different phases. A Mann-Whitney U test was used to compare the effect of mode (GDM versus PCT) in every different phase-target combinations (5 phases, 5x8 cases for ET targets, and 5x4 cases for GT targets). A Friedman ANOVA test was used to detect the effect of phase (PRE, E-EXP, L-EXP, WAS, and FOL) and target (ET versus GT) separately for the two modes. If these effects were statistically significant, further two-by-two comparisons were performed with the Wilcoxon test.

To analyse individual results, we focused on the shoulder abduction/adduction movements. For each subject we compared data from PRE and WAS (the mean final posture of the two movements in PRE with respect to the first two movements in WAS, therefore only during the first repetition of WAS), to determine the presence of after-effects, and data from early EXP and late EXP (the mean final posture of the first two movements in EXP with respect to the last two movements in EXP), to determine the presence of adaptation. On these two intervals, we therefore computed a Student’s t-test.

For the PCA analysis, we computed the distance metric (Section “Principal Component Analysis”) between the subspaces describing the PRE trial and all the subsequent blocks in the same mode. These distance were then compared to subspaces describing the spontaneous variability observed during the spontaneous variability experiment (4 blocks of 5 repetitions). To this purpose, for each mode, we calculated the mean of the permutation of the distance between PCs subspaces and used this value as hypothesized value for a non parametric one-sample sign test.

Finally, correlation analyses were also performed between morphological and kinematic data.

All the statistical computation were performed using Statistica (Ⓒ *Dell Inc.*).

## Results

First the results of the human spontaneous variability within the exoskeleton are presented. Then the outcomes on two illustrative participants are detailed in order to show the effect of KSC, within the ABLE exoskeleton, and the consequent inter-individual difference. Finally, separately, the results of the experiment are analysed with respect to the whole group of participants and the three different tasks, considering successively the effect of the direct exposition to the corrective force fields (movements towards ET), and the consequences over targets and movements which were never exposed to these fields (towards GT).

### Human spontaneous variability

We aimed at measuring the spontaneous human variability and capturing the variability due to the different arrangement of the device on the human arm, consequence of detaching and attaching the participants to the exoskeleton. The outcome of this preliminary experiment showed a small and stable variability among the 10 subjects while moving with the exoskeleton in gravity compensation mode. The effect of the connection/disconnection from the robot also did not affect largely the stability of the results. Visualization of this variability will be showed in the follow of this section, together with the results of the force field exposition experiment, in order to give a reference for comparing with human unperturbed movements.

### Individual results

#### Two illustrative cases

**Fig. 5:**
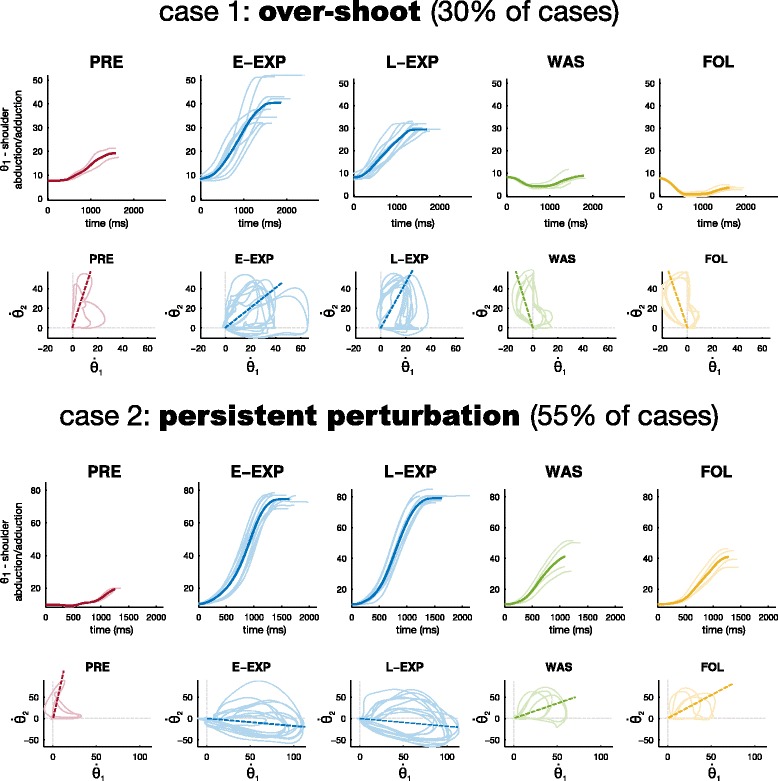
Two illustrative cases. For two subjects, during GDM task, we show two different figures: on *top*, the averaged trajectory of the the shoulder abduction/adduction (dark plots) and the single trajectories (lighter plots), when moving towards ET 4; on *bottom*, for the same target, the resulting cycloids when considering the ratio between the first two joint velocities (shoulder abduction/adduction versus internal/external rotation). In this case the light plots are the cycloids, while the dark dashed lines are the mean ratio. For the four graphs, data refer to the 5 phases of the experiment, preliminary (PRE), early exposition (E-EXP, first five repetitions of EXP), late exposition (L-EXP, last five repetitions of EXP), wash-out (WAS), and follow up (FOL)

Figure 5 is composed of four figures showing the results of two subjects, which represents the different behaviours, observable during the experiment. For both results, the task was the goal-directed mode. The top figure, for each case, represents the trajectories of the first angle (the shoulder abduction/adduction) on motions towards the ET number 4 (placed on the left of the plane including the shoulder, thus producing internal shoulder rotation), for 5 phases of the experiment, respectively preliminary, early exposition (first five repetitions of EXP), late exposition (last five repetitions of EXP), wash-out, and follow up.

As we can see, the effect of the KSC (E-EXP) was, in one situation (case 1) to increase the value of abduction above the initial level (PRE). Then during the continuous exposition to the KSC (L-EXP) the level of abduction tended to return to the original one. After the removal of KSC (WAS), the subject showed an over-shoot of the shoulder angle in the opposite direction of the constraints (in this case, a stronger adduction) which persisted during follow-up. Similar results were observed mainly for the first two angles, on most of the ET positions.

In the other situation (case 2), clearly the post-effect after the exposition of the force fields is rather a coordination in between the one imposed by the KSC and the natural one. In addition we did not see any type of adaptation during the L-EXP phase.

Another way to illustrate these phenomena is by looking at the velocity cycloids i.e. graphs showing the velocity of a joint according to the velocity of another joint, thus showing joint velocity synergies. The bottom graph of Fig. 5, for each case, shows the cycloids for movement towards target number 4 and, in particular, the velocity of the first joint (shoulder abduction/adduction) with respect to the second joint (shoulder internal/external rotation), during the 5 phases of the experiment. The results are consistent with the two phenomenona of either late-exposition adaptation towards the original movement, with a strong over-shoot as post-effect during both WAS and FOL, or absence of adaptation in L-EXP, followed by persistent perturbation as post-effect during both WAS and FOL. These are also underlined by the dashed lines representing the mean ratio between the two joint velocities.

#### Inter-individual differences

During WAS and FOL, most of the movements resulted in either a persistent perturbation or in an over-shoot. In a smaller number of movements, no effects were observed when the KSC was removed. At the same time, generally either adaptation occurred during late-EXP, or participants did not changed their coordination while moving within the force fields. The behaviour of each subject, during and after the exposition to KSC, was identified thanks to the analysis described in Section “Statistics”. Based on these, the persistent perturbation was the most common effect (55% of the cases), while one third of the tasks were followed by over-shoot. No effects were observed only on 22 cases out of 160 (14% of cases), and these were almost equally distributed on the two tasks. GDM resulted mostly in persistent perturbation during WAS (63% of the movements), while during PCT we observed a slightly increase of over-shoot effects (48% of persistent perturbation versus 33% of over-shoot). Finally late exposition adaptation occurred only in the 21% of the movements, more frequently during pointing tasks (GDM) than in tracking (PCT).

### Movements towards ET: adaptation to KSC

#### Joint kinematics

**Fig. 6:**
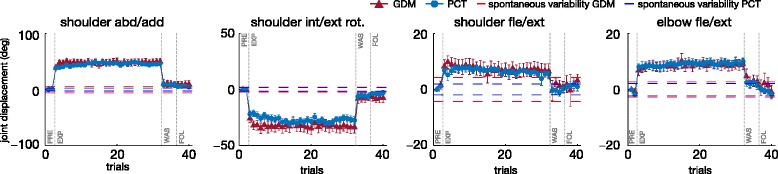
Mean joint final displacement on movement towards ET 3. Mean joint final displacement and standard error with respect to the final posture of first movement in PRE, in the two different modes, over the 10 subjects. The target position is ET number 3. Columns are the four joint of the exoskeleton. Horizontal dashed lines represent the joint maximum standard deviation *σ* for the spontaneous variability experiment

The mean final upper-limb posture, for ET 3 over the whole experiment and for both tasks, is presented in Fig. 6. The illustrated target was chosen since its height and internal position, with respect to the right arm of the participants, involved large rotations on which the effects of the KSC are more observable.

Mann-Whitney U test performed separately for the 160 cases (4 joints, 5 phases, 8 targets combinations) showed that the final angle in the different joints did not vary significantly with the mode except in 4/160 cases (3 times for joint 2, once for joint 4).^1^ Friedman ANOVA, performed separately for each mode, showed that the posture, for all the joints, varied significantly with the targets and the phases (*p*<0.001). Paired comparisons, through the Wilcoxon test, were used to analyze the differences between phases separately for each mode and each target.

First joint angle (shoulder abduction/adduction) increased significantly between PRE and E-EXP, for both GDM and PCT mode, for all the targets (*p*<0.01), indicating the direct effect of the KSC. It remained increased by reference to PRE during L-EXP (*p*<0.01). There were no significant differences between E-EXP and L-EXP phases, except for a slight increase of the deviation for target 4 (*p*<0.01), and a decrease for target 7 in PCT mode (*p*<0.05). The angle returned close to PRE values during WAS for all the targets but for targets 6 in GDM mode, for which the deviation persisted (*p*<0.01). During FOL the joint postures were mostly similar to PRE values, except for targets 6 in both modes (*p*<0.05).

Second joint angle (shoulder internal/external rotation) decreased significantly between PRE and E-EXP (for targets 1 and 3–6, whatever the mode *p*<0.01, for target 8 in GDM and target 7 in PCT *p*<0.05). This deviation lasted during the exposition to the KSC and remained different to PRE values during L-EXP (*p*<0.01), except for targets 2 and 7. There were significant differences between E-EXP and L-EXP phases only for target 5 in GDM (*p*<0.05) and targets 4, 5, and 8 in PCT (*p*<0.05). During WAS, the joint angles remained decreased by reference to PRE values for target 6 in GDM (*p*<0.01) and targets 2 and 5 for PCT. During FOL, the values were decreased by reference to PRE values for targets 3, 5 (*p*<0.05) and 6 (*p*<0.01) in GDM.

Third joint angle (shoulder flexion/extension) was significantly modified by the KSC as shown by significant differences between PRE and E-EXP, except for target 1 (*p*<0.01 for targets 3–7, *p*<0.05 for targets 2,8 in GDM mode and *p*<0.01 for targets 3–8 in PCT mode). The direction of the modification depended on the target: the angle was increased for targets 2–5 and decreased for targets 6–8. During L-EXP, the angle remained deviated in the same direction (*p*<0.05 for targets 3 and 4, *p*<0.01 for targets 5–8 in GDM, *p*<0.01 in PCT). There were only slightly differences between E-EXP and L-EXP depending on the mode and the target.^2^ The amount of the deviation decreased during WAS (without changing direction) but remained significant for targets 6–8 in GDM (*p*<0.01) and for targets 1, 5 (*p*<0.05) and 6, 8 (*p*<0.01) in PCT. During FOL, the values were not significantly different with respect to the PRE phase.

Fourth joint angle (elbow flexion/extension) significantly increased between PRE and E-EXP (*p*<0.01 whatever the mode and the target). This deviation remained during L-EXP, as shown by significant differences between PRE and L-EXP, except for target 7 (*p*<0.01 for targets 2–6, 8 and *p*<0.05 for target 1 in GDM, *p*<0.01 for targets 3–6, 8 and *p*<0.05 for target 1 and 2 in PCT). There were no differences between E-EXP and L-EXP in GDM and only a decrease of the deviation for target 1 in PCT (*p*<0.05). During WAS, the values were not significantly different from the ones in PRE, for the GDM, except for a persisting deviation for target 5 (*p*<0.05). In PCT, there was a persisting deviation for targets 3 (*p*<0.05) and 5 (*p*<0.01), and a reversal of the effect with decreased values, by reference to the PRE values, for targets 1 and 7 (*p*<0.05). During FOL, the values were not different to those in PRE, in GDM, but they remained decreased w.r.t. PRE for targets 1 and 7 in PCT (*p*<0.01).

In conclusion, the KSC consistently modified the final joint postures and its effect lasted as long as the exposition to the perturbation with small differences between the early and late periods. After the removal of the KSC, participants were often performing differently from their natural coordination, depending on the mode and the target. The after-effect were mostly characterized by the persistence (in the same direction but with a reduced amount) of the deviation observed during the exposition to KSC. A significant overshoot was only observed for joint 4, during reaching for two targets in PCT.

#### End-point kinematics

**Fig. 7:**
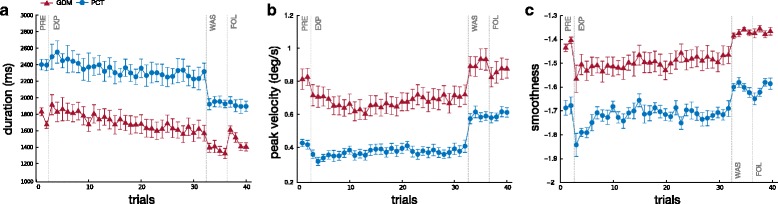
Mean motion duration T (**a**), mean peak velocity *v*_*max*_ (**b**), and mean smoothness *η* (**c**). Averaged data over the ten participants and standard error for the pointing tasks towards ET positions for the two different modes. Smoothness, through spectral arc-length, is higher when *η* is closer to zero

Motion duration and peak velocity for ET motions are shown in Fig. 7 a and Fig. 7 b. Mann-Whitney U test, performed separately for the 40 cases, confirmed that the velocity of the movements was slower in PCT than GDM mode (significant in 36/40 cases, the exceptions were during WAS or FOL) with a longer duration (significant in 29/40 cases, the exceptions were mostly during FOL). Friedman ANOVA performed separately for each mode showed that the velocity and duration varied significantly with the target and the phase (*p*<0.001). Post-Hoc Paired comparison, through the Wilcoxon test, confirmed that the peak velocity decreased during the early exposition to KSC (significant decrease between PRE and E-EXP for 2 targets in GDM mode and 4 in PCT mode), then it remained stable during the exposition to KSC between E-EXP and L-EXP (except a significant increase for one target in PCT). The peak velocity increased after the removal of KSC (significant difference between L-EXP and WAS for six targets in GDM and for every target in PCT, and between L-EXP and FOL, for seven targets in GDM and for every target in PCT). The velocity was slightly higher during FOL than PRE in PCT mode (significant for seven targets). The duration of the movement was slightly increased at the beginning of KSC (significant difference between PRE and E-EXP for two targets for GDM and PCT) then decreased during exposition to KSC (significant difference between E-EXP and L-EXP for four targets in GDM and PCT). The duration decreased further after the removal of KSC (significant difference between L-EXP and WAS for four targets in GDM and five in PCT, and between L-EXP and FOL for one target in GDM and for five in PCT). During WAS and FOL the movements were shorter than before exposition to KSC (significant difference PRE-WAS for five targets in GDM and for all target in PCT, significant difference PRE-FOL for three targets in GDM and six targets in PCT).

As expected (Fig. 7 c), the movements were less smooth in PCT than in GDM. This was confirmed by Mann-Whitney U test showing a significant difference in 32/40 cases. Friedman ANOVA performed, separately for each mode, showed that the smoothness varied significantly with the target and the phase (*p*<0.001). The exposition to the KSC immediately decreased the smoothness of the movement (Wilcoxon: significant difference between PRE and E-EXP for six targets in GDM and two in PCT) and this effect lasted during the KSC exposition (no difference between E-EXP and L-EXP; except one target in PCT). When the KSC was off, the smoothness was slightly improved during WAS by reference to L-EXP (significant difference in four targets in GDM and three in PCT).

**Fig. 8:**
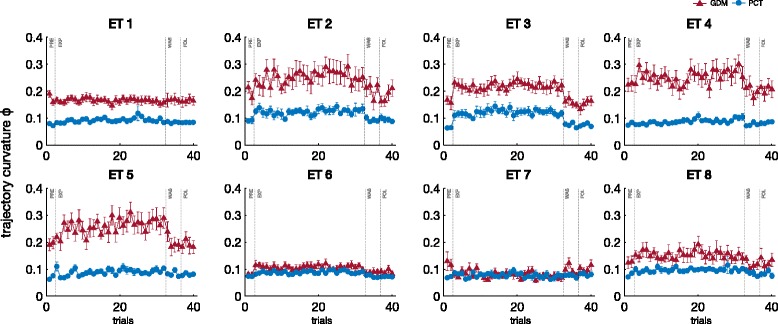
Mean trajectory curvature *Φ*. Mean trajectory curvature and standard error for the ten subjects, over the 8 ET positions, for the two tasks

The variation of the trajectory curvature *Φ* as a function of ET target position for the different modes is shown on Fig. 8. Mann-Whitney U test confirmed that the curvature was usually greater in GDM than in PCT (significant in 27/40 cases). The increase of curvature was observed for the more distant targets (target 1–5 during all the phases and target 8 for phases PRE and E-EXP) but not the closest ones (targets 6 and 7). Friedman ANOVA, performed separately for each mode, showed that the curvature varied significantly with the target and the phase (*p*<0.001). The curvature increased at the beginning of exposition to KSC (significant difference between PRE and E-EXP for targets 3 and 6 in GDM, and targets 2, 3, 6, 8 in PCT) then remained at a similar level during the exposition to KSC (no significant difference between E-EXP and L-EXP, except target 4 in PCT). The curvature regained PRE level during WAS and FOL.

Summarizing, the results from velocity and duration parameters are consistent: the subjects slowed down their movements as soon as the KSC became active. Once the KSC was removed, the movements became faster and shorter. The smoothness of the trajectories was lessened and their curvature increased during the application of the KSC without clear after effects.

#### Inter-joint coordination

**Fig. 9:**
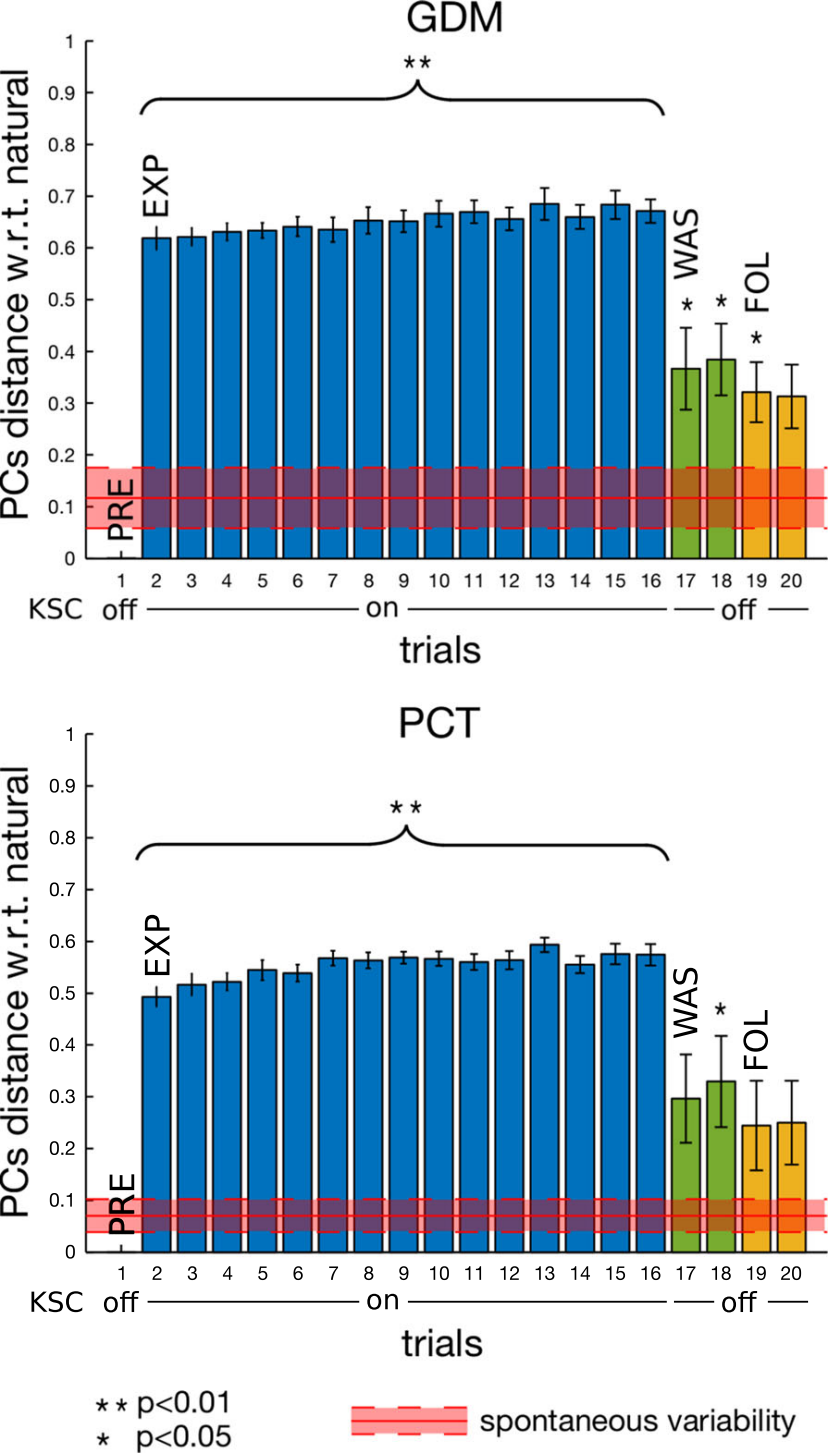
PCs distance from PRE, on ET pointing task, for the two tasks GDM and PCT. PC subspaces mean distance and standard error with respect to first repetition in PRE phase (trial 1) over the 10 participants when pointing toward ET. In red, mean values and standard deviation of spontaneous variability experiment with 5 healthy subjects of Section “Quantification of the human spontaneous variability within the exoskeleton”. Asterisks * mean significant difference w.r.t. spontaneous variability after non parametric one-sample sign test

PCA analysis and the above-mentioned subspace-distance based metrics were used to analyse the effect of KSC on inter-joint coordination. By construction, the KSC should increase the distance of the EXP PCs from the natural PCs (the one computed during the PRE phase) and decrease the distance from the space defined by the KSC constraining vector of Eq. 2. Figure 9 shows the distance between PCs subspaces from the motions in the preliminary phase, thus motions before the exposition to any force fields (the first value of the distance is null, since it represents the distance of the first synergy from itself). For the two tasks, we can clearly see the consistent effect of the presence of the KSC during the experiment phase (bars in blue). In fact these distances are large and almost constant for all the subjects in all the cases (GDM and PCT). Horizontally, in red, we plotted the value of the spontaneous variability experiment showing a visual representation of natural synergy variability of healthy humans performing pointing tasks, see Section “Quantification of the human spontaneous variability within the exoskeleton”.

The last four bars, respectively two for WAS and two for FOL, represent the post-effects of the force fields exposition. During these phases, participants were no longer under the constraints by the KSC, but were instead performing with a gravity compensated robot, similarly to the PRE phase. Wash-out synergies, both in GDM and PCT exercises, show statistically significant difference to the spontaneous variability value (after non parametric one-sample sign test). In path-constrained tracking task, this difference is also kept during the FOL phase, meaning that a different inter-joint coordination was still present, on most of the participants, even 30 minutes after having performed the last movements under the perturbation by the KSC.

**Fig. 10:**
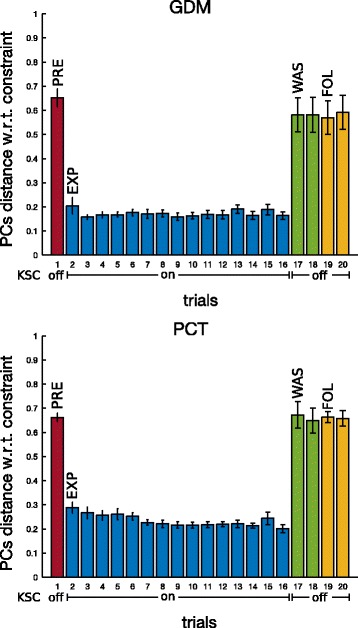
PCs distance from constraining vector, on ET pointing task, for the two tasks GDM and PCT. PC subspaces mean distance and standard error from constraining vector (Eq. 2) (Eq. 2) over the 10 subjects during pointing task towards ET

Figure 10 shows the distance between each PCs subspace with respect to the subspace computed from the constraining vector of Eq. 2. Mainly a small distance indicates that the subject is performing the movement following the imposed constraints, while a large distance stands for different upper-limb patterns of coordination. Therefore the KSC was able to correctly constrain the participants to perform the desired synergy (EXP phase) for each mode. On the other side, the post-effect of WAS and FOL phases does not seem to correspond to the effective constraints on the joint coordination.

### Movements towards GT: catch-trials unexposed to KSC

#### Joint kinematics

Catch-trials to GT were performed with the exoskeleton in gravity compensation mode and the reaching movements towards GT targets have never been exposed to KSC-generated force fields.

Mann-Whitney U test, performed separately for the 80 cases (4 joints, 5 phases, 4 targets combinations), showed that the final angle in the different joints did not vary significantly with the mode except in 7/80 cases (3 times for joint 1, once for joint 2 and 4, and 2 times for joint 3). Friedman ANOVA, performed separately for each mode, showed that in GDM the posture for all the joints varied significantly with the target and the phase (*p*<0.05 for joint 1, *p*<0.001 for the other joints), while, in PCT, it varied significantly with the target and the phase for joint 1 (*p*<0.01), joint 2–4 (*p*<0.001), but not for joint 3.

First joint angle (shoulder abduction/adduction) increased significantly between PRE and E-EXP for targets 1, 2, and 4 (*p*<0.05) in GDM, and for target 3 in PCT. It remained increased, by reference to PRE, during L-EXP for target 1 in GDM (*p*<0.05). The angle returned close to PRE values during WAS and FOL.

Second joint angle (shoulder internal/external rotation) decreased significantly in GDM between PRE and E-EXP for target 2 (*p*<0.05), and between PRE and L-EXP for target 1 (*p*<0.05). A decrease also appeared for target 3 during WAS and FOL by reference to PRE. There were no significant differences in PCT.

**Fig. 11:**
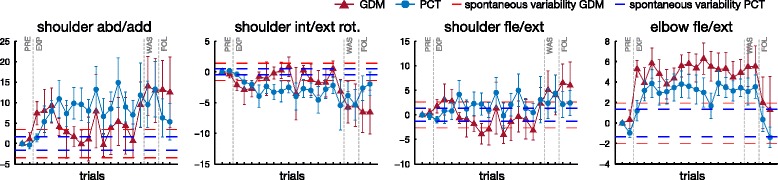
Mean joint final displacement on movement towards GT 3. Mean joint displacement and standard error with respect to final posture in PRE, in the two different mode, over the 10 subjects. The target position is GT number 3. Columns are the four joint of the exoskeleton. Horizontal dashed lines represent the joint maximum standard deviation *σ* for the spontaneous variability experiment

The third joint angle (shoulder flexion/extension), during GDM, was significantly decreased for target 4 between PRE and E-EXP (*p*<0.05) and for targets 1 and 4 between PRE and L-EXP (respectively *p*<0.05 and *p*<0.01). It remained decreased during WAS for target 1 (*p*<0.05). It was also increased for target 3 but not significantly due to large variability (Fig. 11).

Fourth joint angle (elbow flexion/extension) significantly increased between PRE and E-EXP (in GDM: *p*<0.05 for target 1, *p*<0.01 for targets 2–4, and in PCT *p*<0.05 for targets 1, 3, 4). This deviation remained during L-EXP (in GDM for target 3, *p*<0.05, and in PCT for targets 1, 3, *p*<0.05 and 4, *p*<0.01). During WAS, the increase remained significant for target 3 in GDM and for targets 1 and 3 for the PCT. During FOL, the values were not different than in PRE, except a decrease in PCT for target 2 (*p*<0.05).

In brief, during the experimental period when the KSC was active, it also consistently modified the final posture during catch-trials to targets that have never been directly exposed to KSC, suggesting both spatial and temporal generalization of the effect of KSC. This effect depended of the target but lasted as long as the exposition to the force fields. When significant, the after-effects were characterized by the persistence of the deviation observed during the exposition to KSC.

#### End-point kinematics

**Fig. 12:**
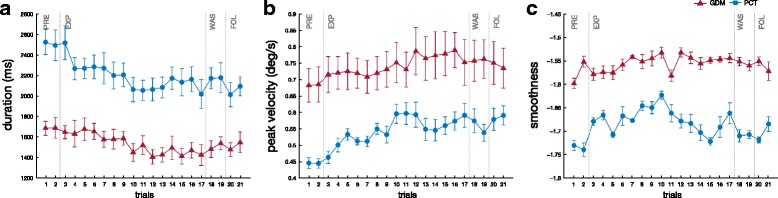
Mean motion duration T (**a**), mean peak velocity *v*_*max*_ (**b**), and mean smoothness *η* (**c**). Averaged data over the ten participants and standard error for the pointing tasks towards GT positions for the two different modes. Smoothness, through spectral arc-length, is higher when *η* is closer to zero

Motion duration and peak velocity for motions toward generalization targets are shown in Fig. 12 a and 12 b. Mann-Whitney U test confirmed that the velocity of the movements was slower in PCT than in GDM, with a smaller velocity peak (significant in 14/15 cases for targets 1–3 and 1/5 case for the closest target 4) and a longer duration (significant in 17/20 phase-targets conditions). Friedman ANOVA, performed separately for each mode, showed that the velocity and duration varied significantly with the target and the phase (*p*<0.001). Post-Hoc Paired comparison, with the Wilcoxon test, confirmed that the peak velocity did not change during the early exposition to KSC (no significant difference between PRE and E-EXP), but it increased progressively during exposition to KSC (significant difference between PRE and L-EXP for target 3 in GDM and targets 1, 3, and 4 in PCT). The peak velocity did not change after the removal of KSC (no significant difference between L-EXP and WAS). In PCT, the peak velocity remained higher during WAS and FOL than during PRE (significant for 4 targets).

The duration of the movement was slightly decreased at the beginning of KSC (significant difference between PRE and E-EXP for target 3 in GDM and target 2 in PCT) then decreased during exposition to KSC (significant difference between E-EXP and L-EXP for target 3 in GDM and 1, 3, 4 in PCT). The duration remained stable after the removal of KSC (no significant difference between L-EXP and WAS, nor between L-EXP and FOL). In PCT, the duration was shorter after than before exposition to KSC (significant difference PRE-WAS and PRE-FOL for 3 targets).

As expected, the movements were less smooth in PCT than in GDM (Fig. 12 c). This was confirmed by Mann-Whitney U test: the differences were significant in 14/15 cases for targets 1, 2, 4 and 1/5 case for target 3. Friedman ANOVA showed that the smoothness varied significantly with the target and the phase in PCT (*p*<0.05), but not in GDM. In PCT, the smoothness was improved during the period of exposition to KSC (Wilcoxon: significant difference between PRE and E-EXP for targets 1, 2 and between PRE and L-EXP for target 1).

**Fig. 13:**
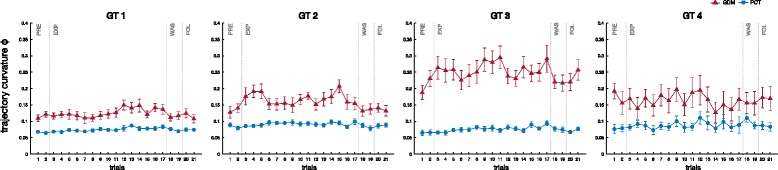
Mean trajectory curvature *Φ*. Mean trajectory curvature and standard error for the ten subjects, over the 4 GT positions, for the two tasks

The variation of the trajectory curvature as a function of CT target position for the different modes is shown on Fig. 13. Mann-Whitney U test confirmed that the curvature was greater in GDM than in PCT for the more distant GTs but less for the closest target 4 (significant difference in 14/15 cases for targets 1–3 and in 1/5 cases for target 4). Friedman ANOVA performed separately for each mode showed that the curvature varied significantly with the target and the phase (*p*<0.001 in GDM, *p*<0.01 in PCT). In GDM, the curvature increased at the beginning of the exposition to KSC (significant difference between PRE and E-EXP for targets 2, 3) and remained at the same level (no significant difference between E-EXP and L-EXP). In PCT, the curvature was significantly increased during the late period (significant difference between E-EXP and L-EXP for targets 1, 3). Then it did not change during the exposition to KSC (no significant difference between E-EXP and L-EXP, except target 4 in PCT) then regained PRE level during WAS and FOL.

Summarizing, in contrast to the direct slowing effect of the KSC, the movements performed during the catch-trials toward generalization targets had a tendency to be performed faster with an improved smoothness. This effect was progressively built up and maximized during the L-EXP period. The effect of KSC on the curvature, observed for the experimental targets, was generalized for the GT targets.

#### Inter-joint coordination

**Fig. 14:**
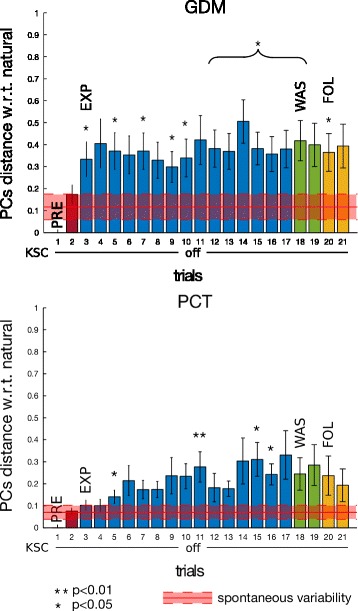
PCs distance from PRE, on GT pointing task, for the two tasks GDM and PCT. PC subspaces mean distance and standard error with respect to first repetition in PRE phase (trial 1) over the 10 participants when pointing toward GT. In red, spontaneous variability mean values and standard deviation of spontaneous variability experiment with 5 healthy subjects of Section “Quantification of the human spontaneous variability within the exoskeleton”. Asterisks * mean significant difference w.r.t. spontaneous variability after non parametric one-sample sign test

**Fig. 15:**
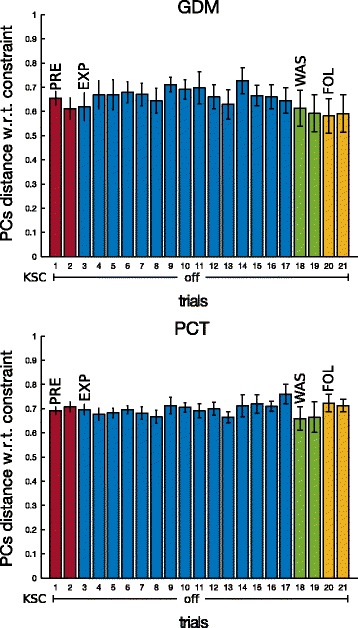
PCs distance from constraining vector, on GT pointing task, for the two tasks GDM and PCT. PC subspaces mean distance and standard error from constraining vector (Eq. 2) over the 10 subjects during pointing task towards GT

PCA analysis showed that the distance between spaces, defined by the 3 main PCs, during the catch-trials progressively increased with respect to the initial coordination, during the EXP phase, and it lasted afterwards, during WAS and FOL, as shown by Fig. 14. A non parametric one-sample sign test performed with respect the spontaneous variability experiment, showed that most of the resulting different synergies, above all in GDM, were statistically significant. The distance between the actual coordination and the constraining vector was not significantly modified (Fig. 15) as previously observed for the movements exposed to KSC.

## Discussion

### Adapting to new upper-limb synergies

**Results overview** Twenty participants of our experiment performed a total of 404 pointing/tracking tasks, 240 of which under the effect of inter-joint perturbing viscous force fields. Subjects were all healthy, naive, without any known pathology OF the upper-limb.

Results of this campaign showed the capability of most of the subjects to learn from this unknown and unnatural environment, meaning that their natural upper-limb coordination, within an ABLE exoskeleton, was exhibiting effects of the force fields exposition in terms of adaptation, post-effects (still visible after a 30 minute rest sessions outside of the robot), and generalization. These results extended the previous conlcusions found on experiments with planar robots [8] or, preliminary, with an exoskeleton [19].

**Poor adaptation during the exposition** We observed adaptation to the original coordination, during late-EXP phase, only on 21% of the testing population. In most of the subjects, we did not observe changes neither in their coordination, nor at the end-effector kinematics, between E-EXP and L-EXP, i.e. they did not adapt by compensating for the external perturbation, as usual in state of the art experiments with planar robot. The absence of joint space adaptation was observed also in the results by Mistry et al. [19], but differently from their experiment, since in our case no adaptation of the end-point movements occurred.

**Post-effects: two observable distinct behaviours** At the same time, distinct post-effects were observed after the removal of the KSC: they consisted, in some cases (30%), in over-shoot on the opposite direction of the natural original coordination – thus similarly to Shadmehr and Mussa-Ivaldi original experiment, but at the joint level, rather then at the end-effector – but the main pattern (55%) consisted of a persistence of the perturbation during the wash-out and even the follow up period. This persistence of the perturbation could be the direct consequence of the CNS not globally optimizing the motor behaviour, but rather tending to repeat suboptimal task-satisfying solutions, because of influenced by motor memory, as described by Ganesh et al. [26]. In the remaining 15% of the cases, we did not observe any significant post-effect.

**Different dynamics, similar results: comparing the two testing modes** When considering the constraint of following a path with the end-effector (thus in PCT), PCA results – meaning the existence of larger PCs deviation from the preliminary motion with respect to the spontaneous variability experiment – are weaker than in GDM, but we can still see, almost always, persistent post-effects in movements towards both ET and GT. At the same time, during wash-out and follow up phases, in both cases most of the participants produced an upper-limb coordination strategy modified with respect to their natural preliminary one (respectively 63% persistent perturbation and 29% over-shoot in GDM, versus 48% and 33% in PCT). These results are interesting considering the different dynamics of the two tasks (PCT generally required slower movements and longer duration). Indeed generally GDM task corresponded to ballistic movements, thus probably mostly driven by feedforward control and offline adaptation, while PCT requested stronger visual feedback to correct online the trajectory, as demonstrated by longer duration, slower velocity, and poorer smoothness.

### Application to rehabilitation/Limits

**Differences from existing studies** A clear difference from Shadmehr and Mussa-Ivaldi original experiments and most of the results described in the introduction of this paper, is linked to the nature of the requested task to the participants. In fact, while in 2D experiments, participants were asked to perform point-to-point reaching task following a straight-line path, thus explicitly requiring to contrast the deviating force fields, in our experiment we never asked for any specific behaviour in terms of coordination. This condition clearly allowed the subjects to voluntary decide to resist the effects of the robot, to follow the constraints imposed by the KSC, or, of course, any possible strategy in between. Additionally, in 2D experiments, the perturbations were applied in the task space, as velocity dependant fields on the end-effector, whilst in this case, applying the velocity dependant force fields at the joint space, the subjects were implicitly constrained while moving and while being focused on the reaching tasks.

**Ability to impose a specific synergy** The main purpose of utilizing a KSC-like strategy with post-stroke patients would be the possibility for the exoskeleton to teach an healthy natural upper-limb coordination, in order to correct common negative and pathological compensations in impaired subjects. Our results did not perform as expected, since after the KSC practice, we did not observe the desired coordination imposed by the force fields. This result, observable in the distance from the constraints of graph 10 and 15, could be related to the only dissipative nature of the version of the KSC that we adopted for this experiment. The original KSC [27] presents also a non-dissipative viscous torque, developed to avoid energy waste by the exoskeleton and to encourage motions which satisfy the desired constraints. In the future we will need to verify if, by adding this assistive term to the control law, we could obtain better performance on the post-practice resulting coordination. Furthermore, it is reasonable to consider that imposing and retaining a non-optimal synergy in healthy subjects is highly challenging. Probably the effects of the exposition to the natural correction by the exoskeleton would achieve better outcomes when modifying impaired pathological synergies in post-stroke survivors.

**Subjects awareness** We observed learning in participants who were not aware – at least at the beginning of the training – of the effects of the control law imposed by the exoskeleton, who were only told to focus on the achievement of the task (pushing the button) rather than on the performance of the motion itself (i.e. the inter-joint coordination). The idea of *implicit learning* of motor control was already analysed by Patton et al. [10] in an experiment with a planar robot, observing a detectable reduction in the washout of the after-effects. Indeed, this phenomenon of long lasting effects seems confirmed in our case. Unfortunately, we did not assess quantitatively the level of awareness of the participants, for example, by using a questionnaire. This “unawareness" of the participants could have also interesting application for neurorehabilitation: we can imagine to train patients on rehabilitative exercises for the upper-limb inter-joint coordination while asking them to play video games, involving end-effector movements, on ad-hoc virtual environments. Post-stroke participants could then focus their attention only on the rewards given by the score of the video games and thus only on the end-effector tasks, rather than being involved in performing particular upper-limb motions and therefore being forced to consider multiple goals for the movement of the joints of the arm. Meanwhile the exoskeleton could impose healthy rehabilitative constraints at the joints of the arm, decreasing the cognitive load on the impaired subjects. Moreover, computing performances for inter-joint coordination could be more complex to judge and qualify in order to produce the rewards-metric, mostly due to the human upper-limb redundancy. Thus considering the feedback to the user from the end-point only, while leaving the robot taking care of the correct joint synergy, could simplify the overall rehabilitative therapy.

**Behind no-adaptation and distinct post-effects** An important inter-individual difference was observed, both at the level of presence of adaptation during the exposition, and at the level of consequent post-effects. At the same time, a smaller but not negligible variability within subjects of these parameters was also found. Personal participants data as weight, height, and grasp force were collected and correlations analysis were performed between these data and the results of the experiment. Learning generally happened when subjects were stronger, taller, heavier, and consequently moving faster, but the difference between any two groups of participants (by either defining the group in which we observed post-effects, and the one in which these effects did not happen, or by taking the group which adapted versus the one which did not adapt, or again, the group which resulted in persistent perturbation versus the one which resulted in over-shoot) were not statistically significant for any of these parameters. The fact that our exoskeleton has fixed-length joint, thus not adjustable to the user physical characteristics, and that the distance between the exoskeleton and the WAM manipulator was not changed with respect to the different height of the participants, may have affected the final results.

### Assessing learning effects with an exoskeleton

A central issue for this experiment was clearly the definition of parameters to assess adaptation, post-effects and generalization occurring during the robotic-aided training. Most of the usual adopted indexes to qualify the presence of these phenomena – in literature it is often shown a sufficient number of kinematic parameters as joint trajectory, end-point trajectory, speed profiles of one or more representative subjects – have been mostly developed for analysing 2D tasks, for which the requested behaviour of the participants was clear (point-to-point movements by following a straight-line). We believe that utilizing PCA analysis helped us to quantify phenomena happening during the motion and not only resulting in different configuration at the end of the movements. However the PCs distance metric was often not enough appropriate in describing the arising adaptation: for example, being an unsigned index, it could not discern the observable different post-effects of Section “Inter-individual differences”. Cycloids seemed to be a fine tool when compared to PCs distance, but their computation produced complex reports to analyse (six cycloids per motion for a 4-DOF robot as ABLE).

Also, it is difficult to translate the PC signification into associated physical interjoint phenomenons (i.e. to determine the joint motions expressed by each synergy/PC), especially since the expression of PCs varied a lot between subjects.

## Conclusions

Using a 4-DOF robotic exoskeleton, we were able to modify more than to teach new upper-limb synergies on twenty healthy subjects, performing pointing and tracking tasks. The peculiarity of our experimental protocol allowed us to observe and analyse different effects of learning – *adaptation* to force field, presence of post-effects, transfer or *generalization* of the adaptation – separately on two sets of pointing targets. Although adaptation, during the exposition to the force fields, did not occur in the majority of the cases, the presence of different after-effects was persistent and observable even after 30 minutes from the last constrained movements, which represents a new result with respect to classical motor adaptation experiments with robots.

One reason of this long lasting effects could be linked to the unexplicit nature of the constraints imposed by the exoskeleton and consequently a decreased involvement of the participants in the process of synergy modification. This result, if confirmed, could have interesting effects on the rehabilitation of post-stroke patients and the creation of intensive therapy. Unfortunately, the chosen number of repetitions for observing a complete wash-out was not always sufficient, thus, in the next future, an effort will be done to study the effective duration of these effects.

Many questions remain still unanswered, above all concerning the subset of participants who did not adapt and did show post-effects during the training with the exoskeleton, and the presence of two distinct adaptive behaviours, not necessary correlated with any measurement performed or with the requested tasks. Additionally, one of this two behaviours seems not to follow the well-established internal model hypothesis, due to the absence of over-shoot in the post-effects phase.

Furthermore an interesting investigation should be performed on the effects on the participants when the upper-limb is detached from the robotic exoskeleton. Could we still observe any effect on their arm synergy with respect to their natural unperturbed strategy before the experiment? This fundamental question affects directly the generalization to ADLs, outside the clinical environment and without the assistance of the robot, and thus deeply concerns KSC or other corrective strategies potentiality on the rehabilitation efficacy.

Finally, we could also verify the reaction of the participants when testing the same protocol on multiple sessions, during successive days, to understand if possibly the post-effects are reinforced and/or anticipated in order to provide a better insight on the property of the human CNS and the role of motor memory.

## Endnotes

^1^ Joint 2, during PRE, for target 6 (*p*<0.02) and, during E-EXP, for targets 1 and 4 (*p*<0.05). Joint 4, during E-EXP, for target 1 (*p*<0.01).

^2^ A decrease of the angle for target 1 (*p*<0.05 in GDM, *p*<0.01 in PCT), a lessening of the initial deviation for target 3 (*p*<0.05 in GDM and PCT) or an increase of the initial deviation (for targets 7 and 8, in GDM *p*<0.05, and for targets 6–8 in PCT mode *p*<0.01).

## Additional files

Additional file 1 A variation of the GDM experiment with ten additional participants. (PDF 251 kb)

## Abbreviations

ET: Experimental target
EXP: Experimental phase
FOL: Follow up phase
GDM: Goal-directed mode
GT: Generalization target
KSC: Kinematic synergy control
PCA: Principal component analysis
PCT: Path-constrained tracking mode
PRE: Preliminary phase
WAS: Wash-out phase
