# Supplementary material for: Modifying upper-limb inter-joint coordination in healthy subjects by training with a robotic exoskeleton
Source: J Neuroeng Rehabil. 2017 Jun 12;14:55. doi: 10.1186/s12984-017-0254-x (PMC5469138; doi:10.1186/s12984-017-0254-x)
Supplement: Supplementary file 2 — A variation of the GDM experiment with ten additional participants. (PDF 251 kb) [file 12984_2017_254_MOESM1_ESM.pdf]

A variation of the GDM experiment with ten additional participants

Supplementary material to *Modifying upper-limb inter-joint coordination in healthy subjects by training with a robotic exoskeleton*

In a preliminary study, an additional group of ten naive participants performed goal-directed movements as in the GDM exercise. However, once pushed the target button, instead of being passively brought back to the starting position by the robotic exoskeleton, they were autonomously moving back to it by using an external virtual reality, showing the current position of the exoskeleton and the starting position. During the return phase, the robot was controlled in gravity compensation mode, thus the force fields were turned off. Due to the difficulty of replacing the arm in the initial posture using virtual visual feedback, the average total time for completing the full experiment increased by 30 minutes.

This solution resulted in a request of effort for the participants, who spent more times into the robotic device without being constrained by any force fields; that is, the ratio between time spent exposed to the force fields, and time spent in the gravity compensated exoskeleton decreased with respect to the GDM.

Apart from this peculiarity, the participants followed exactly the same experimental protocol as the other subjects: same target positions, same phases of the experiment, same number of movement repetitions. The spontaneous variability of movements was also assessed in a subgroup of 5 subjects.

We observed roughly the same mean results than during the GDM task with assisted return, described in the article. The analysis of the behaviour of individual subjects during the post-effect period gave also similar results to those observed during the GDM task.

We hypothesized that both the presence of a *dose-effect* (minor time spent under the force fields w.r.t. total time of the experiment) and a direct wash-out of the effect of the KSC during the return phase of movement, would have caused weaker results than in the case of the GDM. However, even if the results were effectively weaker than in GDM, see figure 1, the difference between the two modes (the GDM and the one using the virtual reality) was not statistically significant, after non-parametric statistics.

In conclusion, this additional experiment confirmed the results of the main experiment on a new group of participant. Results seems to be affected by a dose-effect, due to a shorter relative time of exposition to the force fields with respect to GDM, but this was not evidenced by a statistical comparison between the two cases.

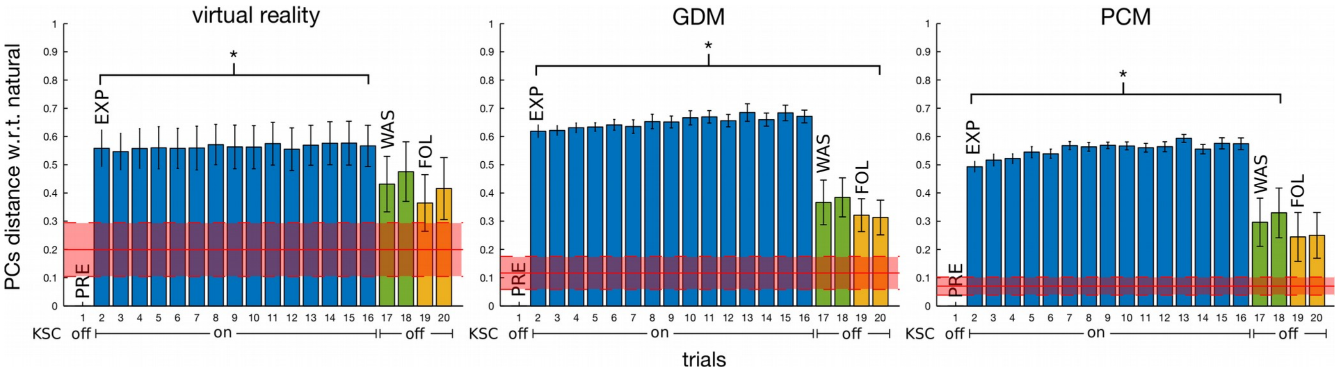

Figure 1: the PCs distance with respect to the movement before the exposition to the fields (the motions in PRE) for the three cases, averaged over the ten participant of each mode, with the associated standard error. The metrics for this distance is explained in the main article. The results for the virtual reality case appeared weaker than for the GDM (due to a larger spontaneous variability, shown by the horizontal red line), but participants were still able to show post-effects during WAS and FOL.
